# Supplementary material for: Chromothripsis during telomere crisis is independent of NHEJ, and consistent with a replicative origin
Source: Genome Res. 2019 May;29(5):737–49. doi: 10.1101/gr.240705.118 (PMC6499312; doi:10.1101/gr.240705.118)
Supplement: Supplemental Material [file supp_gr.240705.118_Supplemental_file_1.zip › contigs/annotated_contigs/DB79/contig.3.DB79_length_593_mean_cov_5.73693086003.docx]

**DB79_length_593_mean_cov_5.73693086003**

```
AATGTATATAAGACATGTGAAGCACTCAAAGGCTAACCGGTATGATGAAGACAGTCATCTGATATGTTGCAGTCACTTGATGGTTCTAG
 >chr9:130158129-130158328 - E=5e-108
CAACCAACATTTTTCAGTCCTGACCACAAGCCTATCCTTAAAGACTTAGGCAACTGCCCTTCTGTACTCCCACAGCATCTAATGCTTGA

TAACTCGAGCACTAGATGTGT|CACATTTGCACACAGAAGTGTGTTAAGTATGAGTT|TATCCTTTGCCCACTTTTTGATGGGGTTGTT
                       >chr9:115761624-115761659 - E=2e-09 p=1e-03  >chr9:123313955-123
TGTTTTTTTCTTGTAAATTTGTTTGAGTTCATTGTAGATTCTGGATATTAGCCCTTTGTCAGATGAGTAGATTGCAAACATTTTCTTCC
314314 - E=2e-198
ATTCTGTAGGTTGCCTGTTCACTCTGATGGTAGTTTATTTTGCACACAGCACTCTTAAGTACATTAGCATGTGTCTCTTTCTTAGGACA

TTTTCCTGTAAAATCATATTATTACCTAATATAAAATTGTTATTCAAGTTTTCCCGGGTATCATCATATGGTTGTTTCTGTAAAAAATT

CAAGATTCTATTGTACTTATTAGTCATGCCTCTTCAACCTCCTTTTATTTAGAAGAGTTCA
```
